# The NICE OECD countries’ geographic search filters: Part 1—methodology for developing the draft MEDLINE and Embase (Ovid) filters

**DOI:** 10.5195/jmla.2021.978

**Published:** 2021-04-01

**Authors:** Lynda Ayiku, Paul Levay, Thomas Hudson

**Affiliations:** 1 lynda.ayiku@nice.org.uk, Information Specialist, Information Services team, National Institute for Health and Care Excellence (NICE), United Kingdom; 2 paul.levay@nice.org.uk, Information Specialist, Information Services National Institute for Health and Care Excellence (NICE), United Kingdom; 3 thomas.hudson@nice.org.uk, Information Specialist, Information Services, National Institute for Health and Care Excellence (NICE), United Kingdom

**Keywords:** search filter, literature searching, MEDLINE, Embase, Geography

## Abstract

**Objective:**

There are no existing validated search filters for the group of 37 Organisation for Economic Co-operation and Development (OECD) countries. This study describes how information specialists from the United Kingdom's National Institute for Health and Care Excellence (NICE) developed and evaluated novel OECD countries’ geographic search filters for MEDLINE and Embase (Ovid) to improve literature search effectiveness for evidence about OECD countries.

**Methods:**

We created the draft filters using an alternative approach to standard filter construction. They are composed entirely of geographic subject headings and are designed to retain OECD country evidence by excluding non-OECD country evidence using the NOT Boolean operator. To evaluate the draft filters’ effectiveness, we used MEDLINE and Embase literature searches for three NICE guidelines that retrieved >5,000 search results. A 10% sample of the excluded references was screened to check that OECD country evidence was not inadvertently excluded.

**Results:**

The draft MEDLINE filter reduced results for each NICE guideline by 9.5% to 12.9%. In Embase, search results were reduced by 10.7% to 14%. Of the sample references, 7 of 910 (0.8%) were excluded inadvertently. These references were from a guideline about looked-after minors that concerns both OECD and non-OECD countries.

**Conclusion:**

The draft filters look promising—they reduced search result volumes while retaining most OECD country evidence from MEDLINE and Embase. However, we advise caution when using them in topics about both non-OECD and OECD countries. We have created final versions of the search filters and will validate them in a future study.

## INTRODUCTION

### NICE and OECD country evidence

**Figure 1:**
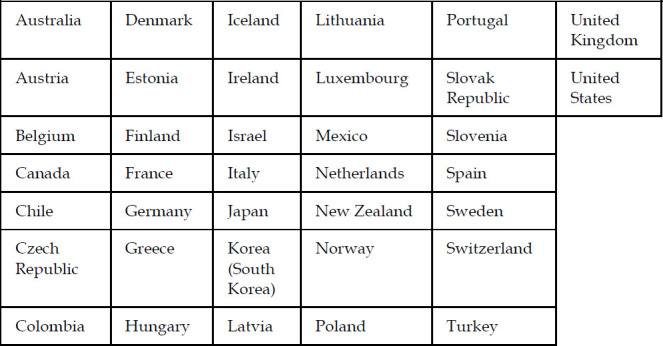
Thirty-seven OECD countries

The Organisation for Economic Co-operation and Development (OECD) is an international agency that works in collaboration with member countries to develop policies for the reduction of inequality and poverty in all nations [1]. OECD members must be democratic and have open, transparent, free-market economies [2]. There are currently 37 OECD country members (Figure 1).

The National Institute for Health and Care Excellence (NICE) in the United Kingdom (U.K.) produces evidence-based national guidance and advice with the aim of improving health and social care [3]. There are nearly 200 NICE guidelines on a variety of clinical, public health and social care topics. The guidelines provide evidence-based recommendations on preventing or managing specific conditions and on planning services and interventions to improve the health of communities [3]. They are used to advise health and social care professionals on how to manage people in their care and deliver services. Evidence about the U.K. and similar countries is often required to inform NICE guideline recommendations. OECD countries are often used as a proxy for countries with similarities to the U.K. [4–8].

### Geographic search filters

Search filters are collections of pre-made, reusable search terms that are applied to bibliographic database literature searches to retrieve evidence with a common feature [9]. Search filters are different from search strategies because their recall (also known as “sensitivity”) has been tested and validated against a gold standard (GS) set (also known as a “reference set”) of known relevant records [10]. Recall is the proportion of references that a filter retrieves from the GS set [10]. The recall figure provides users with an indication of how well the filter works in retrieving the type of evidence they require [10].

Geographic search filters aim to retrieve evidence about geographic locations, such as continents or countries. The InterTASC Information Specialists’ Sub-Group (ISSG) Search Filter Resource is updated regularly through high-quality systematic searches of relevant literature to identify filters [11]. As of December 2020, validated filters for three geographic areas were available in the resource:

1. Africa: PubMed and Embase [12]
2. Spain: PubMed [13]
3. U.K.: MEDLINE [14] and Embase [15]

We previously developed and validated the NICE U.K. geographic search filters for MEDLINE and Embase (Ovid) [14, 15]. These filters retrieve evidence about the U.K. effectively and efficiently when needed for context-sensitive NICE guidance topics [14–16]. They have been used to inform U.K.-focused topics in NICE guidelines [17–19] as well as national reports and systematic reviews produced by external organizations [20–22].

### Requirement for OECD country search filters

The ISSG search filter resource [11] contains an untested search strategy for OECD countries, [23] but no validated search filters for OECD countries are available. This means that geographic limits are not applied to literature searches for NICE guidelines when OECD country evidence is required. The search process for identifying evidence about OECD countries was time-consuming and inefficient. For example, the NICE guideline on tuberculosis [5] required only OECD evidence as an inclusion criterion. However, because validated filters for OECD countries were not available, no geographic restrictions were applied to the search strategy, and the relevant references had to be selected from all the search results.

### Alternative approach to search filter construction for the draft NICE OECD countries search filters

Due to the large number of OECD country members, it would be challenging to develop an effective search filter following the “third generation” search filter development approach, in which search terms are derived objectively through word frequency analysis on a set of relevant references [10]. Instead, the “second generation” search filter development approach was employed to draft the OECD countries filters subjectively, using knowledge about the subject [10].

Creating a geographic search filter for OECD countries that includes free-text terms would be difficult and time-consuming. From our experience gained from developing the NICE U.K. filters [14, 15] plus information from the Spain filter [12], a geographic filter with free-text terms would need to include the following:

- free-text search terms for the country name in the title, abstract, journal name and author institution/correspondence address search fields;
- free-text search terms for all the city names for the country in the title, abstract and author institution/correspondence address search fields;
- free-text search terms for the country's national or regional health services; and
- relevant language variations for the free-text country, city, and health service search terms.

As a pragmatic solution to the challenges of creating filters for OECD countries, we adopted the structure of the “humans” limit for the draft filters as a solution to the challenges of using free-text terms. The humans limit was devised by Carol Lefebvre to exclude results about animals and retain results about humans and was originally appended to a search filter for systematic reviews [24]. The limit was created for the MEDLINE bibliographic database and comprises National Library of Medicine (NLM) Medical Subject Heading (MeSH) terms alone. The limit works by excluding MEDLINE records for animal studies that are indexed with only the “animal” MeSH term using the NOT Boolean operator [24]. It retains MEDLINE records that are indexed with both the human and animal MeSH terms in addition to retaining records that are not indexed with either the human or animal MeSH terms [24].

Since its creation, the humans limit has been adopted widely in literature searches and modified in relation to updates made to MeSH terms and searching functions in MEDLINE. For instance, the Cochrane Highly Sensitive Search Strategies for identifying randomized trials in MEDLINE contain an updated version of the limit [25]. The humans limit has also been translated for use in other databases. The limit is applied to search strategies using the NOT Boolean operator: NOT ([animal subject headings] NOT [human subject headings]).

A limitation of using subject headings alone in the humans limit is that references that are about animals but that are not indexed with subject headings for animals are retained in the search results. However, it would be inadvisable to add free-text terms to the limit because relevant references about humans could be inadvertently excluded if omissions in terms about humans are made. Using subject headings alone reduces the risk of relevant references being missed.

In March 2020, we created draft NICE OECD countries filters to examine the feasibility of the humans limit approach for retaining OECD country evidence and excluding non-OECD evidence. The aim of our study is to improve the effectiveness of literature searches when evidence about OECD countries is required. Our objectives are to develop geographic search filters that retrieve evidence about OECD countries effectively and validate the search filters. We will also share the search filters with information professionals, researchers and health professionals and advise on their use.

In this paper we focus on objective 1.

## METHODS

### Geographic subject headings

Following the humans limit structure [24], the drafts comprise a set of geographic subject heading terms (MeSH in MEDLINE and Emtree in Embase) for OECD countries plus related subject headings and a separate set of geographic subject headings that were available for the non-OECD countries [1]. In our study, non-OECD countries are defined as those that are not members of the OECD.

The draft filters contain subject headings for 36 of the 37 OECD country members. The filters were drafted in March 2020, and Colombia did not become an OECD member until April 2020 [1]. We included Colombia in the non-OECD country subject headings in the drafts.

We created the draft MEDLINE filter first. To identify individual geographic subject headings, we “exploded” the Geographic Locations/MeSH term to reveal the narrower headings beneath. We selected subject headings for the 36 OECD countries in addition to relevant geographic regions that include the countries (for example, Australasia for Australia and New Zealand). Where relevant, we opted for efficiency by exploding geographic subject headings to capture subject headings for the OECD countries simultaneously. For example, ‘exp “Scandinavian and Nordic countries"/’ was used to retrieve subject headings for Denmark, Finland, Iceland, Norway and Sweden. We combined the subject headings with the OR Boolean operator.

We also added further subject headings of relevance to OECD countries. The MeSH terms “Organisation for Economic Co-Operation and Development"/, “Developed Countries”/and “European Union”/were selected and combined with the other OECD country MeSH terms using the OR Boolean operator.

Next, we identified MeSH terms for non-OECD countries from the narrower headings beneath the Geographic Locations/MeSH subject heading. These headings were combined with the OR Boolean operator.

When the draft MEDLINE filter was complete, we created the draft Embase filter. The Geographic Names/Emtree term was “exploded” and the same process was used to select OECD and non-OECD country headings.

We found the identification of OECD and non-OECD country subject headings to be complex in some cases. For instance, some geographically remote overseas territories are linked to OECD countries. Examples include French Guiana, which is an overseas region of the French Republic, and Gibraltar, which is on overseas territory of the United Kingdom [26, 27]. The relevance of evidence from territories such as these was unclear. We decided to omit subject headings such as these from the exclusion line of the filters to ensure that references about these territories were retained.

### Exclusion of irrelevant non-OECD country evidence

Following the humans limit structure [24], the draft NICE OECD countries filters must be applied to literature search strategies using the NOT Boolean operator. This contrasts with the usual use of the AND Boolean operator to apply search filters to search strategies with the aim of retrieving evidence directly. We used the NOT Boolean operator to exclude evidence about non-OECD countries. We structured the draft NICE OECD filters as follows:

* NOT ([‘Non-OECD country’ subject heading terms] NOT [‘OECD country’ subject heading])

The draft NICE OECD filters aim to do the following:

- exclude database records that are indexed only with subject headings for non-OECD countries;
- retain database records that are indexed only with OECD country subject headings;
- retain database records that are indexed with both OECD and non-OECD country subject headings; and
- retain records that are not indexed with either OECD or non-OECD country subject headings.

**Figure 2:**
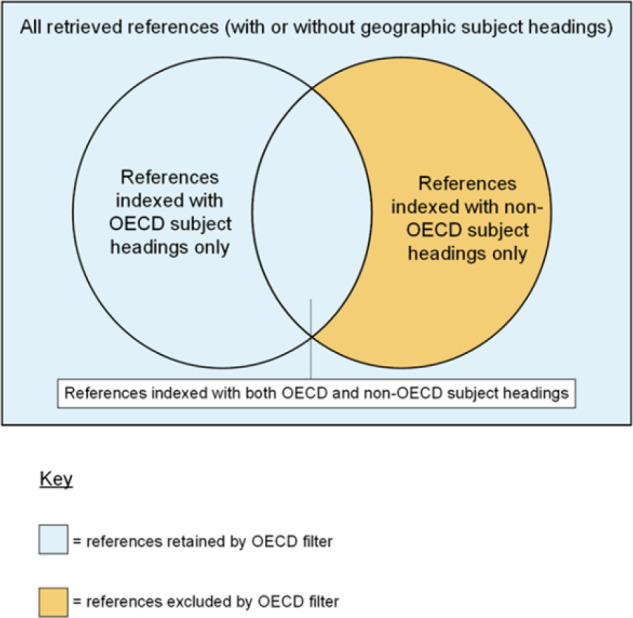
Venn diagram showing the conceptual structure of the OECD countries filters (not to scale)

The Venn diagram in Figure 2 illustrates the concepts above.

### Evaluation of the search filters

We applied the draft filters to MEDLINE and Embase (Ovid) literature search strategies for the following three NICE guidelines:

- NICE guideline 1: Indoor air quality at home [28]
- NICE guideline 2: Looked after children and young people (in development) [29]
- NICE guideline 3: Social, emotional and mental wellbeing in primary and secondary education (in development) [30]

We chose the literature search strategies for these guidelines because they retrieved >5,000 results in both the MEDLINE and Embase databases, and we believed the large search results would provide useful sets to test the ability of the filters to exclude non-OECD country references and retain OECD country references. The MEDLINE literature search strategies can be found in Appendix 1, and the Embase literature search strategies can be found in Appendix 2.

On March 31 and April 1 of 2020, we re-ran the MEDLINE and Embase literature search strategies for each of the guidelines and applied the relevant draft filter to each of the strategies. The MEDLINE filter was applied to the MEDLINE strategies and the Embase filter was applied to the Embase strategies. We calculated and recorded the percentage of results that were excluded by the filters by comparing the number of results retrieved by the original strategies with the number of results retrieved when the filters were applied to the strategies.

To evaluate the search filters’ effectiveness at excluding non-OECD country evidence, the lead author screened samples of 10% of the excluded MEDLINE and Embase database records for each guideline to determine whether OECD country results had inadvertently been excluded by the draft filters. To generate a representative selection of records, the lead author downloaded and imported the first 10 records from sets of 100 records (e.g., 1–10, 101–110, 201–210, etc.) into EPPI-Reviewer software. The lead author screened the title, abstract, and subject heading fields of the sample database records in EPPI-Reviewer to determine whether they contained details about OECD countries. The lead author marked records that contained OECD country details in EPPI-Reviewer.

### Finalization of the search filters

We have updated the draft filters for the validation phase of the project (see Appendix 3). The OECD countries section of the filters now includes Colombia, which joined the OECD in April 2020, and Costa Rica, which is in the process of joining [1]. In addition, we amended the structure of the geographic subject headings. Subject headings for all the individual OECD and non-OECD countries have now been included (some broad headings had been previously exploded to capture individual countries).

## RESULTS

**Figure 3:**
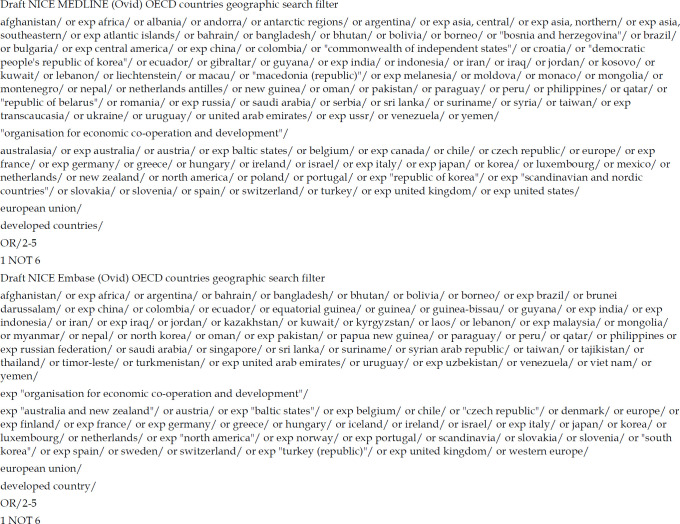
Draft NICE OECD countries geographic search filters

Our draft NICE OECD countries geographic search filters for MEDLINE and Embase (Ovid) can be found in Figure 3, with final versions available in Appendix 3.

### MEDLINE results

The draft MEDLINE filter reduced result volumes by 12.4% for NICE guideline 1, by 9.5% for NICE guideline 2, and by 12.9% for NICE guideline 3 (Table 1). Details of the MEDLINE literature search strategies, including number of search results, can be found in Appendix 1.

**Table 1:** Draft NICE MEDLINE OECD countries filter results

NICE guideline topic	No. of results from MEDLINE search strategy	No. of results from MEDLINE search strategy with draft MEDLINE filter applied	Reduction	No. of sample MEDLINE records	No. of sample MEDLINE records about OECD countries that were excluded inadvertently
NICE guideline 1: Indoor air quality at home	11,842	10,370	1,472 (12.4%)	150	0
NICE guideline 2: Looked after children and young people	14,687	13,288	1,399 (9.5%)	140	2
NICE guideline 3: Social, emotional and mental wellbeing in primary and secondary education	11,328	9,759	1,569 (12.9%)	160	0

Overall, we examined 450 database records from the 4,440 excluded MEDLINE results, and no records about OECD countries were identified for NICE guidelines 1 and 3 (Table 1). For NICE guideline 2, “Looked after children and young people,” two records (1.4%) of relevance to an OECD country were identified [31, 32]. These references were about the international adoption of minors from China by adoptive parents based in the United States [31, 32]. All the references used for the MEDLINE evaluation are available in Appendixes 4 and 5.

### Embase results

The draft Embase filter reduced result volumes by 12.5% for NICE guideline 1, by 10.7% for NICE guideline 2, and by 14.0% for NICE guideline 3 (Table 2). Details of the Embase literature searches and search result numbers can be found in Appendix 2.

**Table 2:** Draft NICE Embase OECD countries filter results

NICE guideline topic	No. of results from Embase search strategy	No. of results from Embase search strategy with draft Embase filter applied	Reduction	No. of sample Embase records	No. of sample Embase records about OECD countries that were excluded inadvertently
NICE guideline 1: Indoor air quality at home	16,719	14,622	2,097 (12.5%)	210	0
NICE guideline 2: Looked after children and young people	13,183	11,770	1,413 (10.7%)	150	5
NICE guideline 3: Social, emotional and mental wellbeing in primary and secondary education	6,879	5,913	966 (14.0%)	100	0

We examined 460 database records from the 4,476 excluded Embase results, and no records about OECD countries were identified for NICE guidelines 1 and 3 (Table 2). We found five records of relevance to an OECD country (3.3%) within excluded Embase results for NICE guideline 2 [33–37]. These references were about the international adoption of minors from non-OECD countries by adoptive parents based in OECD countries or about unaccompanied asylum seeker minors from non-OECD countries in OECD countries [33–37]. All the Embase references used for the evaluation are available in Appendixes 6 and 7.

## DISCUSSION

Our findings show that the draft NICE OECD geographic countries search filters for MEDLINE and Embase exclude non-OECD evidence and retain the vast majority of OECD country evidence. Our draft filters reduced search results by 9.5% to 12.9% in MEDLINE and by 10.7% to 14% in Embase. This is important because screening references during evidence selection processes for evidence-based products such as guidelines and systematic reviews is time-consuming. The Cochrane Handbook, for example, estimates that 500–1,000 abstracts can be screened in an 8-hour period [38]. Using this calculation, our results show that the draft search filters equated to a saving of approximately two days spent on evidence selection. This could ultimately reduce guideline and systematic review development times when OECD country evidence is required, because fewer irrelevant geographic search results means that time spent on selecting evidence (plus associated resource costs) is reduced.

Once validated, the filters could be used to exclude evidence about a variety of geographic regions beyond OECD country distinctions because the filters are composed of country subject headings. For instance, for research topics that require a broader criteria for developed nations (such as the inclusion of the BRICS countries [Brazil, Russia, India, China, and South Africa] [40] or World Bank nations [41]), searchers could transfer subject headings from the non-OECD countries section of the filter to the OECD countries section to retain the additional relevant geographic results. Similarly, for research topics about low-and middle-income countries (LMICs), the order of the geographic subject headings in the filters could be reversed to exclude search results about OECD countries and other high-income nations. Although the retrieval effectiveness for this has not yet been examined, we plan to test the potential to modify the filters to retrieve evidence about other geographic regions during a later phase of the project.

### Next steps

The final draft filters will be validated using a gold standard (GS) set in accordance with search filter development methodology [9, 10]. The GS set will be generated via the relative recall method [39] and will contain references about OECD countries that have informed NICE guidelines. The final draft filters will then be validated by calculating their recall against the GS set references. When complete, our NICE OECD countries search filters will be the first validated geographic filters to be composed of subject headings and make exclusive use of the NOT Boolean operator to exclude irrelevant evidence and retain relevant evidence.

There are currently only three validated geographic search filters for the U.K., Africa and Spain [12–15]. We hope that this work on geographic search filters will encourage the development of additional validated search filters for more locations around the world [42].

## LIMITATIONS

Using geographic subject heading terms alone for the draft filters has limitations. For instance, not all references are indexed with geographic subject headings [12–16]; therefore, some irrelevant non-OECD country evidence will be retained. However, this limitation does not pose risks for OECD evidence to be excluded inadvertently.

The draft filters did not exclude any database records that were focused solely on an OECD country. However, a small number of references (7 out of 910 records; 0.8%) about looked-after minors from non-OECD countries who now live in OECD countries were excluded [31–37]. This is because only the geographic subject headings for the non-OECD countries had been assigned in the database records. For this reason, we advise that the draft filters should be used with caution for topics that focus on both OECD and non-OECD countries, such as international adoption or migration.

We screened approximately 10% of excluded MEDLINE and Embase database records for each guideline to determine whether OECD country references had been excluded by the draft filters by mistake; it is therefore possible that some of the unscreened database records may have included OECD country references. In addition, only the lead author screened the references, and it is possible that alternative selection choices could have been made if the records had been screened by all three authors.

A further limitation is the potential time required to update the geographic search filters to reflect changes in OECD country memberships, MeSH and Emtree OECD and non-OECD country terms, and changes to the names of the countries. However, as the filters comprise subject headings, updating the filters should be a straightforward task.

## CONCLUSION

The novel draft NICE OECD countries geographic search filters for MEDLINE and Embase look promising. The draft filters retain most of the evidence about OECD countries and could potentially save time for guideline development when OECD country evidence is required. However, they should be used with caution for topics that focus on both OECD and non-OECD countries.

We plan to validate the final versions of the filters in the next phase of this study. When complete, the NICE OECD countries geographic search filters will be the first validated filters to be composed of subject headings and make exclusive use of the NOT Boolean operator with the aim of excluding irrelevant evidence to retain relevant evidence.

## Data Availability

Data associated with this article (Appendixes 1–7) are available at https://osf.io/9a7ug/.
